# Sanitation of Cement for Fillings, Crowns and Bridges

**Published:** 1907-09-15

**Authors:** M. J. Emelin

**Affiliations:** New York, N. Y.


### SANITATON OF CEMENT FOR FILLINGS, CROWNS AND BRIDGES.

BY M. J. EMELIN, D. D. S., NEW YORK, N. Y.

Every dental practitioner knows how dreadful and shocking to the sense of smell is the odor of crowns or bridges just removed from a patient who has worn them for any length of time. All crevices, pits and air spaces, as between the porcelain facings and the gold, are filled with stagnant moisture, very detrimental to the healthy condition of an oral cavity and its contiguous tissues.

Exposed fillings suffer less disintegration than cements confined in roots or crowns, and it is common knowledge that cements disintegrate under the influence of the oral fluids, giving rise to a filthy condition. Often a person, sensitive to cleanliness of the mouth, readily detects the foul odor by means of a toothpick or floss silk, and then consults his dentist. As claimed, the predominating factor of such decomposition is the oxyphosphate liquid; and to remedy this evil I recommend the use of solidified formaldehyde, which I have been using for about six months. From a number of simple experiments I notice that this preparation, in the above form, is not detrimental to the working qualities of any good cement, except that it slightly hastens the setting, and in consequence more heat is evolved. In fact, I am inclined to believe that the edge-strength of some cements is improved. I believe that under ordinary conditions the solidified formaldehyde, properly incorporated will keep the cement indefinitely sweet. I have used it with equally good results in filling root canals, with cement and campho-phenic powder, in proportion of 1 to 4. The quantity of solidified formaldehyde for devitalized teeth is larger than that for teeth in vitality.

During the short experimental period I have observed no complications.

To further improve the sanitary conditions of a bridge, crown or any other denture which is to give service in the mouth, I apply paraffin invariably. The dentures should be dry and just warm enough to cause the paraffin to melt gently into all crevices and air spaces, filling them out completely. The paraffin I use is previously sterilized by melting it with a few crystals of solidified formaldehyde. Crowns of bridges I pickle cold in strong acids, wash in solution of bicarbonate of soda, and finish in the usual way. When ready for cementation, I place on a slab the solidified formaldehyde, cement powder and liquid. I combine the drug with the liquid oxyphosphate, and, after the thorough incorporation of both, I add the cement powder and proceed as usual.

At this point it is well to bear in mind that the solidified formaldehyde quickens the setting of a mix. I would suggest the use of an agate, platinoid or German silver spatula. The quantity of the formaldehyde used for cementation of one crown is equal to size of a head of a round burr No. 4 S. S. White. I find that the same quantity of the drug is also useful for cement fillings.—*Dental Brief.*

